# MGA Mutation as a Novel Biomarker for Immune Checkpoint Therapies in Non-Squamous Non-Small Cell Lung Cancer

**DOI:** 10.3389/fphar.2021.625593

**Published:** 2021-04-13

**Authors:** Lei Sun, Man Li, Ling Deng, Yuchun Niu, Yichun Tang, Yu Wang, Linlang Guo

**Affiliations:** Department of Pathology, Zhujiang Hospital, Southern Medical University, Guangzhou, China

**Keywords:** MGA, mutation, non-squamous non-small cell lung cancer, biomarker, immune checkpoint inhibitors

## Abstract

**Background:** Immune checkpoint inhibitors have changed the treatment landscape for advanced non-small cell lung cancer. However, only a small proportion of patients experience clinical benefit from ICIs. Thus, the discovery of predictive biomarkers is urgently warranted. Evidence have shown that genetic aberrations in cancer cells can modulate the tumor immune milieu. We therefore explored the association between oncogenic mutations and efficacy to ICIs in non-squamous NSCLC.

**Methods:** We curated genomic and clinical data of 314 non-squamous NSCLC patients receiving ICIs from four independent studies for the discovery cohort. For external validation, 305 patients from an ICI-treated cohort and 1,027 patients from two non-ICI-treated cohorts were used. Relations between oncogenic mutations and outcomes of immunotherapy were examined. Multivariate Cox regression models were applied to adjust confounding factors. Further investigation on tumor antigenicity and antitumor immunity was performed in The Cancer Genome Atlas lung adenocarcinoma cohort.

**Results:** A total of 82 oncogenes/tumor suppressor genes according to the Oncology Knowledge base database with a frequency greater than 3% were identified and investigated in the discovery cohort. Within these genes, MGA mutations were enriched in patients with durable clinical benefit (*p* = 0.001, false discovery rate *q* < 0.05). The objective response rate was also significantly higher in patients with MGA mutation (2.63-fold, *p* < 0.001, FDR *q* < 0.05). Longer progression-free survival was found in MGA-mutated patients (HR, 0.41; 95% CI, 0.23–0.73; *p* = 0.003), and the association remained significant after controlling for tumor mutational burden (TMB), programmed cell death ligand-1 expression, and treatment regimens. In the validation cohort, significant improvement in overall survival was found in patients harboring MGA mutation (HR, 0.39; 95% CI, 0.17–0.88; *p* = 0.02). Furthermore, the survival difference was not detected in non-ICI-treated cohorts. We also demonstrated that MGA mutation correlate with higher TMB, elevated neoantigen load and DNA damage repair deficiency. Gene set enrichment analysis revealed that gene sets regarding activated immune responses were enriched in MGA-mutated tumors.

**Conclusion:** Our work provides evidence that MGA mutation can be used as a novel predictive biomarker for ICI response in non-squamous NSCLC and merits further clinical and preclinical validation.

## Introduction

Immune checkpoint inhibitors (ICIs) targeting programmed cell death (ligand)-1 (PD-1/PD-L1) and/or cytotoxic T lymphocyte-antigen 4 (CTLA-4) have emerged as a promising treatment for patients with advanced non-small cell lung cancer (NSCLC) (Lee et al., 2018; Garon et al., 2019; Hellmann et al., 2019). Despite their remarkable success, clinical benefit only occurs in a small subset of patients with NSCLC. Therefore, there is an increasing interest in investigating biomarkers for predicting response to ICIs, both for enabling precision medicine and better understanding the mechanisms of resistance.

A variety of biomarkers have been developed to identify cancer patients who would benefit from ICIs, such as PD-L1 expression (Topalian et al., 2012; Gibney et al., 2016), tumor mutational burden (TMB) (Yarchoan et al., 2017; Hellmann et al., 2018), microsatellite instability (MSI) status (Le et al., 2017), and tumor-infiltrating lymphocytes (TILs) (Tumeh et al., 2014; Sade-Feldman et al., 2018). However, the clinical utility of these biomarkers has been limited for various reasons, including spatial intratumor and intertumoral heterogeneity, inconsistent measuring methods, lack of standardized cut-off value, and relatively high cost. Thus, there is a critical need to identify novel and reliable predictive biomarkers for checkpoint inhibitor-based immunotherapy.

A growing evidence indicates that activation of oncogenes or loss of tumor suppressor genes may enhance or dampen the immune system (Wellenstein and de Visser, 2018). Oncogenic mutations also correlate with antitumor immunity and response to immunotherapy. STK11 mutation in lung adenocarcinoma patients is associated with “cold” tumor immune microenvironment, and it predicts poor anti-PD1 response (Koyama et al., 2016; Skoulidis et al., 2018). Studies show that mutations in TP53 were associated with an increased PD-L1 expression and co-occurrence of KRAS and TP53 mutation could predict response to PD-1 blockades in lung adenocarcinoma (Dong et al., 2017). In addition, genomic alterations in antigen presentation and interferon-gamma (IFN-γ) signaling pathways were found to be associated with resistance to ICIs (Gao et al., 2016; Zaretsky et al., 2016). These findings suggest that exploring mutations of oncogenes and tumor suppressor genes may be useful for patient stratification.

Here, by collecting and consolidating the genomic and clinical data of non-squamous NSCLC patients treated with ICIs from five published studies (Rizvi et al., 2015; Hellmann et al., 2018; Miao et al., 2018; Rizvi et al., 2018; Samstein et al., 2019), we systematically explored the association between oncogenic mutations and efficacy to ICIs in non-squamous NSCLC. Significantly, our results show that MGA mutations are specifically enriched in patients with durable clinical benefit (DCB) after immunotherapy, strongly associated with higher objective response rate (ORR), improved progression-free survival (PFS), and longer overall survival (OS). This work provides evidence that MGA mutation may serve as a novel predictive biomarker of response to ICIs in non-squamous NSCLC.

## Materials and Methods

### Clinical Cohorts

To evaluate the relationship between recurrently mutated genes and patient response to ICIs, we collected data of ICI-treated NSCLC patients from previously published articles (Rizvi et al., 2015; Hellmann et al., 2018; Miao et al., 2018; Rizvi et al., 2018; Samstein et al., 2019). The discovery cohort includes 314 non-squamous NSCLC patients from four independent cohorts with annotated response data to checkpoint inhibitor therapy. The first cohort (Rizvi et al., 2018) comprised of 206 advanced non-squamous NSCLC patients enrolled from Memorial Sloan Kettering Cancer Center (MSKCC) and treated with anti-PD-(L)1 monotherapy or in combination with anti-CTLA-4. The second cohort (Hellmann et al., 2018) was part of CheckMate-012 clinical trial, comprising 59 non-squamous NSCLC patients treated with combined PD-1 and CTLA-4 blockade. The third (Rizvi et al., 2015) and fourth cohort (Miao et al., 2018), which was curated and filtered by Miao et al. (Miao et al., 2018), included 49 non-squamous NSCLC patients from KEYNOTE-001 clinical trial and the Dana-Farber Cancer Institute. The validation cohort (Samstein et al., 2019) was composed of 305 non-squamous NSCLC patients enrolled from MSKCC, this study provided OS outcomes instead of response data. To access for a general possible association between MGA mutation and patient OS, we also used The Cancer Genome Atlas (TCGA) lung adenocarcinoma cohorts (TCGA-LUAD, *n* = 509) (Campbell et al., 2016) and MSKCC non-ICI lung adenocarcinoma cohort (*n* = 416) (Jordan et al., 2017; Zehir et al., 2017). Somatic mutation files and clinical information of all these studies were obtained from cBioPortal (Cerami et al., 2012).

### Clinical Outcomes

The clinical outcomes were DCB (durable clinical benefit), ORR, PFS, and OS. ORR was defined as the proportion of patients achieving complete response (CR) or partial response (PR) according to RECIST v1.1 (Eisenhauer et al., 2009). DCB was defined as CR/PR or stable disease (SD) that lasted more than 6 months; no durable benefit (NDB) was defined as progression of disease (PD) or SD that lasted no more than 6 months (Rizvi et al., 2018). Patients with less than 6 months of follow-up and had not progressed were categorized as not evaluable (NE). PFS was defined as the time from the start of immunotherapy administration to the date of disease progression or death. For the ICI-treated cohort, OS was calculated from the treatment start date. For the TCGA cohort, OS was accessed from the date of first diagnosis and in the MSKCC non-ICI cohort, survival was measured starting from the date of the procedure to obtain the sequenced specimen. Patients who did not die were censored on the last date of follow-up. For survival analysis, patients with survival duration less than 30 days were excluded.

### Association of MGA Mutation With Tumor Antigenicity

In order to access the relationship between MGA mutation with tumor antigenicity, we compared TMB, neoantigen load and defect of DNA damage repair (DDR) pathways between MGA-mutated samples with MGA wild type samples. TMB was calculated as the total number of somatic nonsynonymous mutations in exonic regions of tumor genome examined. Patients enrolled from MSKCC were profiled by MSK-IMPACT panels, which has been proved to accurately estimate TMB (Rizvi et al., 2018). To normalize TMB across panels of different sizes, the total number of nonsynonymous mutations was divided by the coding region target territory, covering 0.98, 1.06, and 1.22 megabases (Mb) for the 341, 410, and 468-gene panels, respectively (Rizvi et al., 2018). Other cohorts conducted whole-exome sequencing (WES) and TMB was normalized by dividing 38 Mb as the estimate of the exome size. Neoantigen load data of TCGA-LUAD cohort were obtained from Thorsson et al. (Thorsson et al., 2018), which was defined as the total number of predicted neoantigens. Gene sets associated with DDR pathways were obtained from Knijnenburg et al. (Knijnenburg et al., 2018). We compared the amounts of nonsynonymous mutations in DDR pathway genes according to MGA mutation status in the TCGA-LUAD cohort, MSKCC validation cohort, and MSKCC non-ICI treated cohort.

### Relationship Between MGA Mutation and Tumor-Infiltrating Immune Cells.

We used the online analytical platform CIBERSORTx (Steen et al., 2020) to analyze the relative abundance of 22 types of tumor-infiltrating immune cells. CIBERSORTx is the next generation version of CIBERSORT (Newman et al., 2015), a deconvolution algorithm which outperformed other methods for characterizing cell composition of bulk tissues. We also download the Chen LUAD cohort (Chen et al., 2020) with whole-exome and transcriptome sequencing data (*n* = 169) from cBioPortal and applied CIBERSORTx analysis.

### Gene Set Enrichment Analysis

In order to investigate biological pathways correlated with MGA mutation, we performed GSEA analysis (Subramanian et al., 2005). The Deseq2 R package was used for differential expression analysis of count data. We used these statistics as input to R-function in ClusterProfile package to do GSEA. The Molecular Signatures database (MSigDB) hallmark gene sets (Liberzon et al., 2015), representing major biological processes, were selected as the reference gene sets. The threshold was set at *p* < 0.05 and false discovery rate (FDR) *q* < 0.1.

### Statistical Analysis

Fisher’s exact test was used to examine the associations between gene mutations with patient responses (DCB vs. NCB, ORR, CR/PR vs. PD), and the FDR based on Benjamini-Hochberg (BH) method was used for multiple comparison correction. To analyze OS and PFS, survival curves were plotted using the Kaplan-Meier method, and the survival differences between subgroups was compared using the log-rank test. The Cox proportional hazards model was used to analyze the effect of potential factors on patient survival, in both univariable and multivariable analyses. The differences of TMB, neoantigen load, DDR gene mutation frequencies and tumor-infiltrating leukocytes between MGA-mutated and wild type tumors were analyzed with Mann-Whitney U test. All statistical tests were two-sided and the significance level was set at 0.05. The statistical analyses were conducted in R (version 3.6.0).

## Results

### Patient Characteristics

**FIGURE 1:**
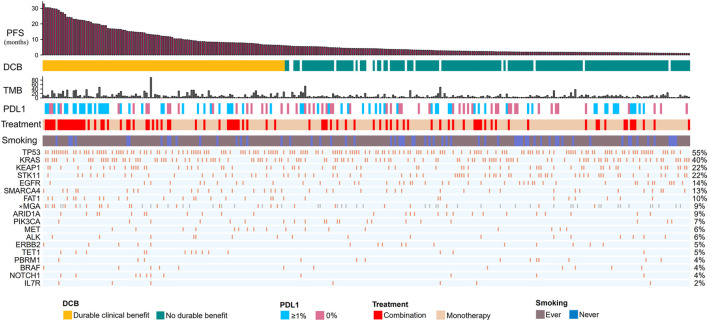
Summary of mutational and clinical information of non-squamous NSCLC patients in the discovery cohort. Individual patients are represented in each column, organized by response category, with progression-free survival time in decreasing order. Categories of smoking status (never or ever) and treatment regimens (combination or monotherapy) are characterized. PD-L1 expression is stratified as 0% or greater than 1%. The occurrences of selected genes in each case are represented in the OncoPrint, with the percent frequency shown. *Mutational information unknown (not covered in the panel tested) are depicted in light gray on the OncoPrint. NSCLC, non small cell lung cancer; DCB, durable clinical benefit; NDB, no durable benefit; PD-(L)1, programmed cell death -(ligand)1; TMB, tumor mutation burden.

Our discovery cohort included 314 non-squamous NSCLC patients receiving ICIs (Figure 1; Supplementary Table S1). These patients were treated with anti-PD-1/anti-PD-L1/anti-CTLA-4 (*n* = 224), or a combination of anti-CTLA-4 and either anti-PD-1 or anti-PD-L1 therapies (*n* = 90). The median age was 64.0 years (22.0–92.0 years). In total, 79% of patients were smokers. Seventy-seven patients (24.5%) had CR/PR; 113 (36%) patients had DCB. The median TMB was 5.70 mutations/Mb (range, 0.184–91.8 mutations/Mb). PD-L1 expression was available for 129 patients, of whom 70 (54%) had greater than 1% expression. Most cohorts did not provide data about ethnicity.

### MGA and TET1 Gene Mutations Were Enriched in Patients Responding to ICIs

**FIGURE 2:**
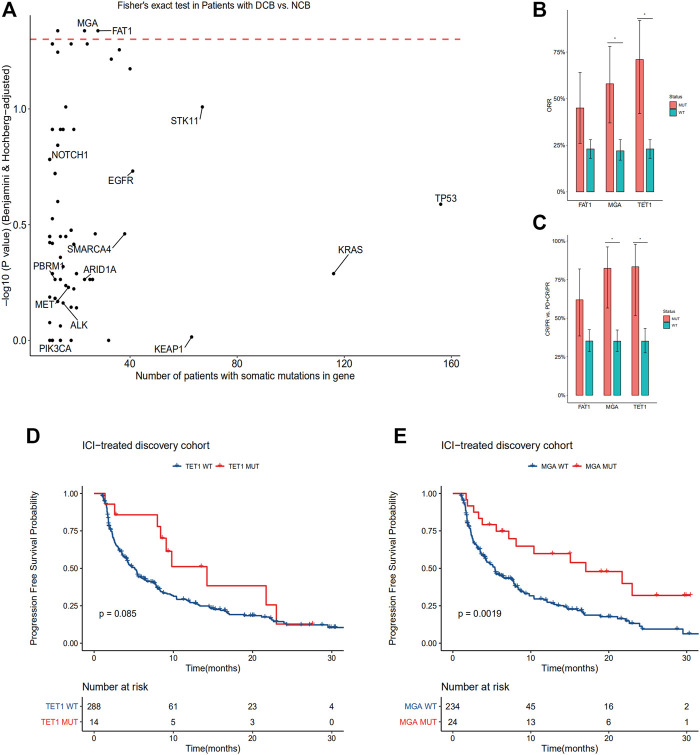
MGA mutation correlate with DCB, higher ORR and longer PFS in the discovery cohort of non-squamous NSCLC patients treated with ICIs **(A)** Enrichment of gene mutations in patients with DCB vs. NCB in the discovery cohort (two-tailed Fisher’s exact test, *n* = 113 patients with DCB, *n* = 181 patients with NCB). Red dashed line denotes BH FDR *q* = 0.05 **(B)** ORR were compared between subgroups according to mutational status of FAT1, TET1 and MGA. *p < 0.05, Fisher’s exact test, BH FDR *q* < 0.05 **(C)** Ratio of patients with CR/PR to patients with PD classified by FAT1, TET1 and MGA mutations. *p < 0.05, Fisher’s exact test, BH FDR *q* < 0.05 **(D–E)** Kaplan-Meier curves comparing PFS of patients with or without TET1 **(D)** and MGA **(E)** mutations in the discovery cohort. A two-sided log-rank test *p* < 0.05 is considered as a statistically significant difference. FDR, false discovery rate; BH, Benjamini-Hochberg method; DCB, durable clinical benefit; ORR, objective response rate; PFS, progression-free survival.

In order to identify statistically robust associations with response to immune checkpoint therapy, we only include oncogenes and tumor suppressor genes according to Oncology Knowledge base (OncoKB) database (Chakravarty et al., 2017) with a frequency greater than 3% in the discovery cohort. A total of 82 genes were identified and investigated (Supplementary Table S2). Within these genes, mutations in MGA, TET1 and FAT1 were enriched in patients with durable clinical benefit (*p* < 0.05, Fisher’s exact test, BH FDR *q* < 0.05, Figure 2A). MGA and TET1 gene mutations were also enriched in patients with CR or PR (Figures 2B,C; Supplementary Figure S1). The ORR of patients with TET1 mutation is 71%, compared with 23% in patients with TET1 wild type. The ORR of patients with MGA mutation is 58% (95% CI 37–78%), compared with 22% (95% CI 17–28%) in patients with MGA wild type. The differences between FAT1 mutated type and wild type was not significant either for ORR or CR/PR vs. PD + CR/PR (Figures 2B,C).

Consistent with previous studies, mutations in TP53 and POLE were enriched in patients with CR or PR, while mutations in EGFR were enriched in patients with PD (*p* < 0.05, Fisher’s exact test, BH FDR *q* < 0.05 for all genes) (Supplementary Figure S1).

A phase I study of NSCLC patients showed that the response rate was better with anti-PD1 plus anti-CTLA4 therapy than anti-PD1 therapy (Hellmann et al., 2017). And the FDA approved the combination of nivolumab (anti-PD1) plus ipilimumab (anti-CTLA4) as a first-line treatment for metastatic NSCLC patients with positive PD-L1. So we investigated whether treatment regimens would influence patient response to ICIs. The results showed that non-squamous NSCLC patients receiving combination therapy were more significantly likely to obtain DCB (*p* < 0.001, Fisher’s exact test). In addition, the objective response rate was 33% (95% CI, 23–44%) with combination therapy vs. 21% (95% CI, 16–27%) with monotherapy.

Then we conducted a subgroup analysis based on different treatment regimens to see whether the enrichment of MGA or TET1 mutations was still observed in patients with DCB. While MGA mutation was not associated with response to ICIs in the monotherapy group, MGA mutation occurred exclusively in patients with DCB in the combination treatment subgroup (*n* = 10; *p* = 0.001, Fisher’s exact test). On the other hand, while TET1 mutation was not associated with response to ICIs in the combination treatment subgroup, it occurred exclusively in patients with DCB in the monotherapy group (*n* = 6; *p* < 0.001, Fisher’s exact test).

### MGA is an Independent Prognostic Biomarker

In the discovery cohort, we compared PFS of patients according to TET1 or MGA mutational status. PFS is significantly improved in patients with MGA mutation. However, survival difference between subgroups according to TET1 mutational status was not significant (Figure 2D). The median PFS was 21.7 months (95% confidence interval (CI), 12.09 to not reached) in MGA-mutated group vs. 5.4 months (95% CI, 4.18–7.56) in the group of MGA wild type (Figure 2E).

Baseline characteristics were examined according to MGA mutational status, and no significant differences were found between the two groups except for TMB (Supplementary Table S4). We also compared co-mutated oncogenes and tumor suppressor genes according to MGA mutational status, but none of them pass FDR correction of 10%.

After adjusting for TMB, PD-L1 expression and treatment regimens by multivariate Cox regression analysis, we found that mutation in MGA was an independent prognostic biomarker of patient survival (*p* = 0.037, hazard ratio (HR) = 0.42, 95% CI 0.19–0.95; Table 1). Besides MGA mutation, PD-L1 expression and treatment regimen were also independently associated with patient survival (Table 1).

**TABLE 1:** Univariate and multivariable Cox regression analysis of progression-free survival in discovery cohort.

Variable	Univariate analysis			Multivariate analysis		
	HR	95% CI	*p* value	HR	95% CI	*p* value
Age (≥65 vs. <65 years)	1.15	0.84 to 1.54	0.37	—	—	—
Gender (male vs. female)	1.13	0.84 to 1.53	0.43	—	—	—
Smoking (ever vs. never)	0.79	0.54 to 1.15	0.22	—	—	—
TMB (high vs. low)	0.87	0.64 to 1.18	0.37	—	—	—
PD-L1 (≥1%vs. 0%)	0.53	0.34 to 0.83	0.006	0.63	0.40 to 1.00	0.049
Treatment (combination vs. monotherapy)	0.69	0.49 to 0.96	0.026	0.57	0.36 to 0.90	0.016
MGA (mutated vs. wild type)	0.41	0.23 to 0.73	0.003	0.42	0.19 to 0.95	0.037

CI, confidence interval; HR, hazard ratio.

**FIGURE 3:**
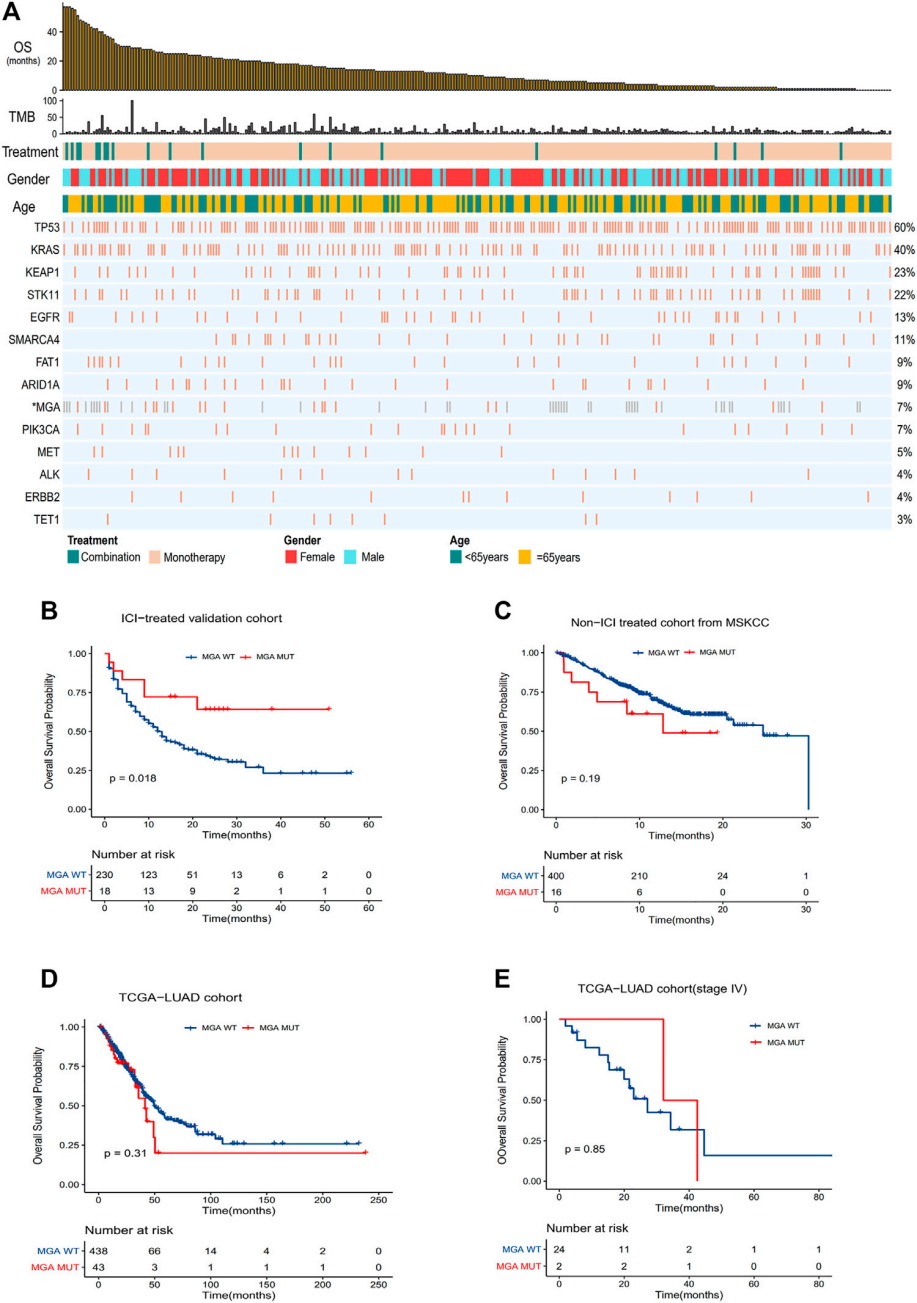
Validation of the predictive function of MGA mutation **(A)** Summary of mutational and clinical information of non-squamous NSCLC patients in the validation cohort. Individual patients are represented in each column, organized by response category, with progression-free survival time in decreasing order. Categories of smoking status (never or ever) and treatment regimens (combination or monotherapy) are characterized. The occurrences of selected genes in each case are represented in the OncoPrint, with the percent frequency shown. *Mutational information unknown (not covered in panel tested) are depicted in light gray on the OncoPrint **(B–E)** Kaplan-Meier curves comparing OS of patients with or without MGA mutations in the validation cohort **(B)**, Non-ICI-treated cohort **(C)**, TCGA-LUAD cohort **(D)** and TCGA-LUAD cohort with stage IV patients **(E)**. Log-rank test was used in **(B–E)**.

To further validate the predictive function of MGA mutation, we compared OS in the validation cohort (Figure 3A). The result showed that OS was significantly improved in patients with MGA mutation (*HR* = 0.39, *p* = 0.02), with the median OS not reached in the MGA-mutated group vs. 12.0 months (95% CI, 10.0–16.0) in the wild type group (Figure 3B). To confirm that the survival benefits observed in patients with MGA mutation were not simply attributed to its general prognostic effect, we further analyzed the survival differences between MGA-mutated and MGA wild type patients in the non-ICI-treated MSKCC cohort and the TCGA-LUAD cohort. There were no significant differences in OS (Figures 3C,D) or PFS (Supplementary Figure S2A) between subgroups according to MGA mutational status. As most patients in the TCGA-LUAD cohort was at early stage, we also compared OS and PFS of patients with stage IV in the TCGA-LUAD cohort. There was no significant difference (Figure 3E, Supplementary Figure S2B).

We also compared OS in the validation cohort according to TET1 genotype. Although there was a trend toward favorable prognosis in patients with TET1 mutation, the survival difference was not significant. A previous study have found that TET1 mutation can serve as a pan-cancer biomarker to ICI response (Wu et al., 2019). We also observed that TET1 mutation was enriched in patients responding to ICIs and correlated with higher ORR. But our results showed no statistically significant improvement in PFS and OS in non-squamous NSCLC patients harboring TET1 mutation. The mutation frequency of TET1 in non-squamous NSCLC patients was around 3%. The relative small sample size may have limited the power to detect significant associations. Thus a larger study is needed to study the relationship between TET1 mutation and efficacy to ICIs in non-squamous NSCLC patients.

The mutation frequency of MGA was 6.3 and 7.8% respectively in ICI-treated cohorts and the TCGA-LUAD cohort. The somatic mutation sites of the MGA gene were randomly distributed, without any 3D hotspot mutation annotations (Cerami et al., 2012) (Supplementary Figure S3).

### MGA Mutation is Associated With Enhanced Antigenicity

**FIGURE 4:**
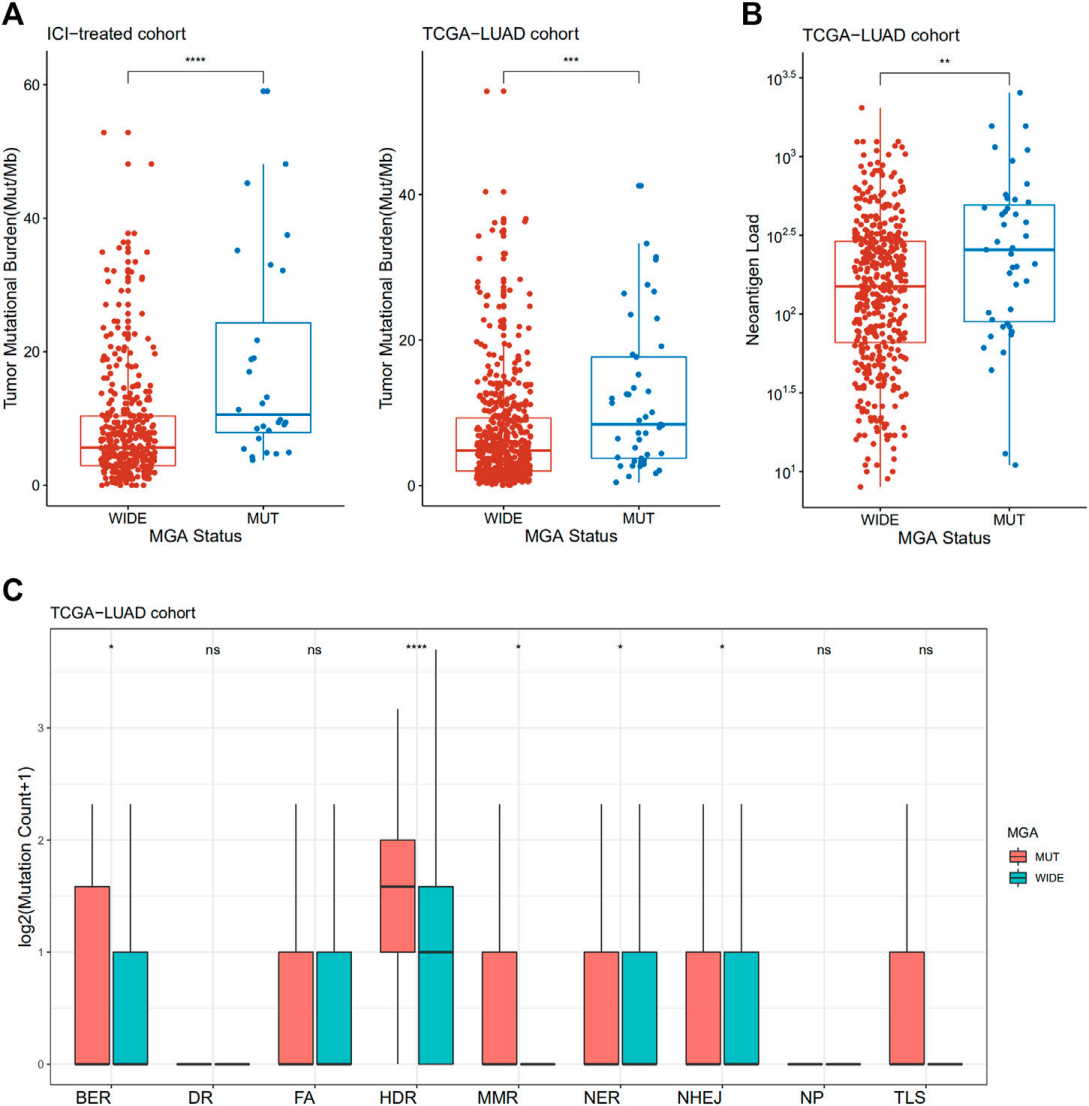
Association of MGA Mutation with tumor mutational burden, neoantigen load and DNA damage repair (DDR) deficiency in patients with non-squamous NSCLC **(A)** Comparison of tumor mutational burden between MGA-mutated and wild-type subgroups from ICI-treated NSCLC and the TCGA-LUAD cohorts **(B)** Comparison of neoantigen load between MGA-mutated and wild-type subgroups in the TCGA-LUAD cohort **(C)** Comparison of mutation amounts of DDR pathway genes between MGA-mutated and wild-type subgroups in the TCGA-LUAD cohort. Mann-Whitney *U* test was used to test the differences. ns: not significant; *p < 0.05; **p < 0.01; ***p < 0.001; ****p < 0.0001. BER, base excision repair; DR, direct damage reversal repair; FA, Fanconi anemia; HDR, homology-dependent recombination; MMR, mismatch repair; NER, nucleotide excision repair; NHEJ, non-homologous end joining; NP, nucleotide pool maintenance; TLS, translesion DNA synthesis.

To investigate the underlying mechanism linking MGA mutation to ICI response, we compared the TMB level and neoantigen load between tumors with MGA wild type and MGA mutation. Non-squamous NSCLC samples with MGA mutations had a significantly higher level of TMB in both the ICI-treated cohort and TCGA-LUAD cohort (*p* < 0.001, Figure 4A). We also observed that MGA-mutated cancers harbor significantly higher neoantigen load compared to samples with MGA wild type (*p* < 0.01, Figure 4B).

Defects in DDR system leads to genome instability, which in turn increase the overall rate of somatic mutations, so we investigated whether MGA mutation is correlated with DDR deficiency. We observed an enrichment of DDR gene mutations in MGA-mutated samples. In the TCGA-LUAD cohort, tumors with MGA mutations had a significantly increased number of DDR pathway mutations, including base excision repair (BER), homology-dependent recombination (HDR), mismatch repair (MMR), nucleotide excision repair (NER), and non-homologous end joining (NHEJ) (Figure 4C). Patients enrolled from MSKCC were profiled by MSK-IMPACT panels, so many DDR genes were not covered. We analyzed DDR pathways as a whole in MSKCC ICI-treated validation cohort and MSKCC non-ICI treated cohort. Although not statistically significant, there was a trend toward more DDR gene mutations for MGA-mutant patients in MSKCC ICI-treated validation cohort (Supplementary Figure S4A). In the MSKCC non-ICI treated cohort, DDR gene mutations were significantly increased in MGA-mutant patients (Supplementary Figure S4B).

### Impact of MGA Mutation on Immune Infiltration and Immune Response

**FIGURE 5:**
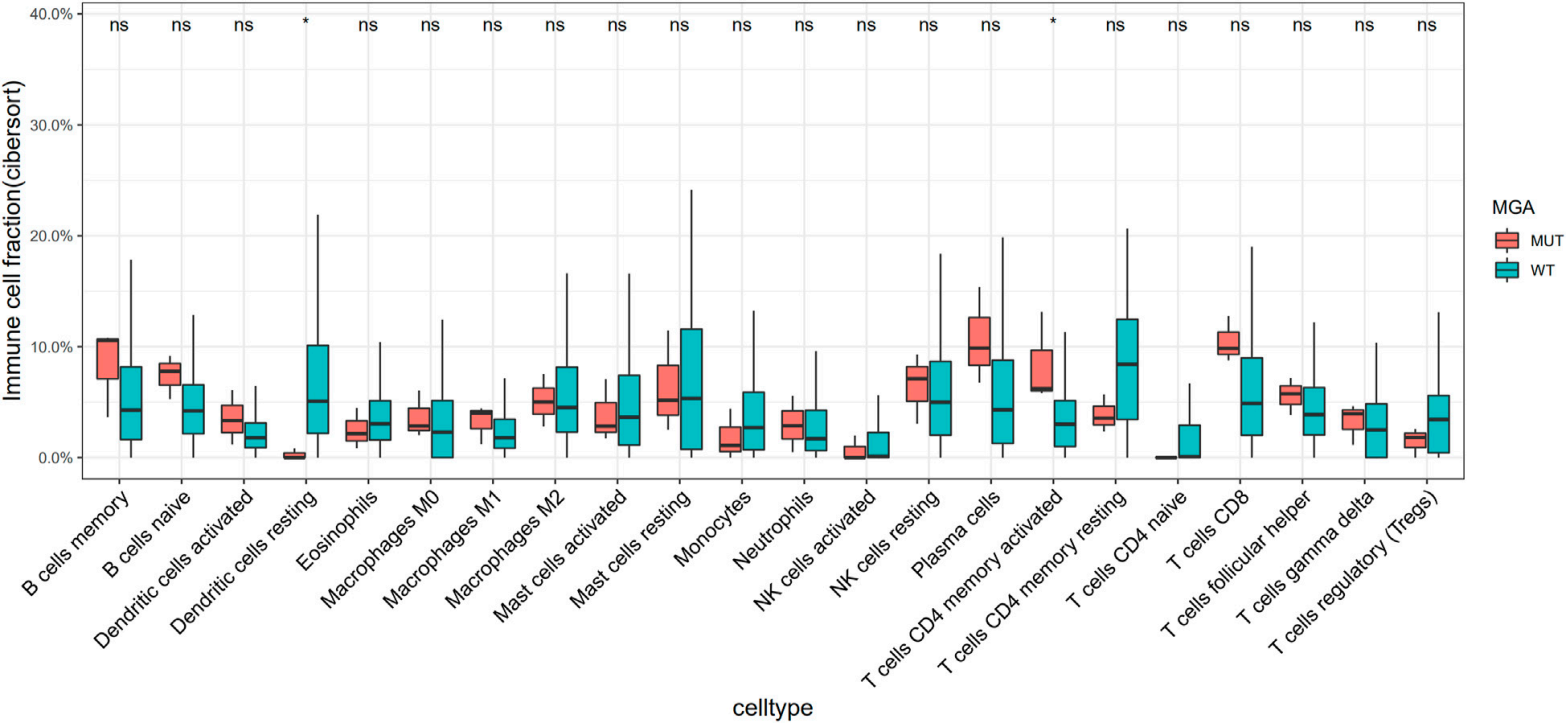
Association of MGA Mutation with relative abundance of infiltrated immune cell by CIBERSORTx in the Chen cohort. Gene expression data were uploaded to the CIBERSORTx web portal (https://cibersortx.stanford.edu/), with batch correction performed and permutation number setting to1000 for significance analysis. *p < 0.05 (Mann-Whitney *U* test).

Using Cibersortx, we did not find any higher level of immune cells infiltration in MGA-mutated tumors compared with wild-type ones in the TCGA-LUAD cohort (Supplementary Figure S5). However, we explored the Chen cohort (Chen et al., 2020) and observed that activated CD4 T cells were more enriched in MGA mutate type group (Figure 5).

**FIGURE 6:**
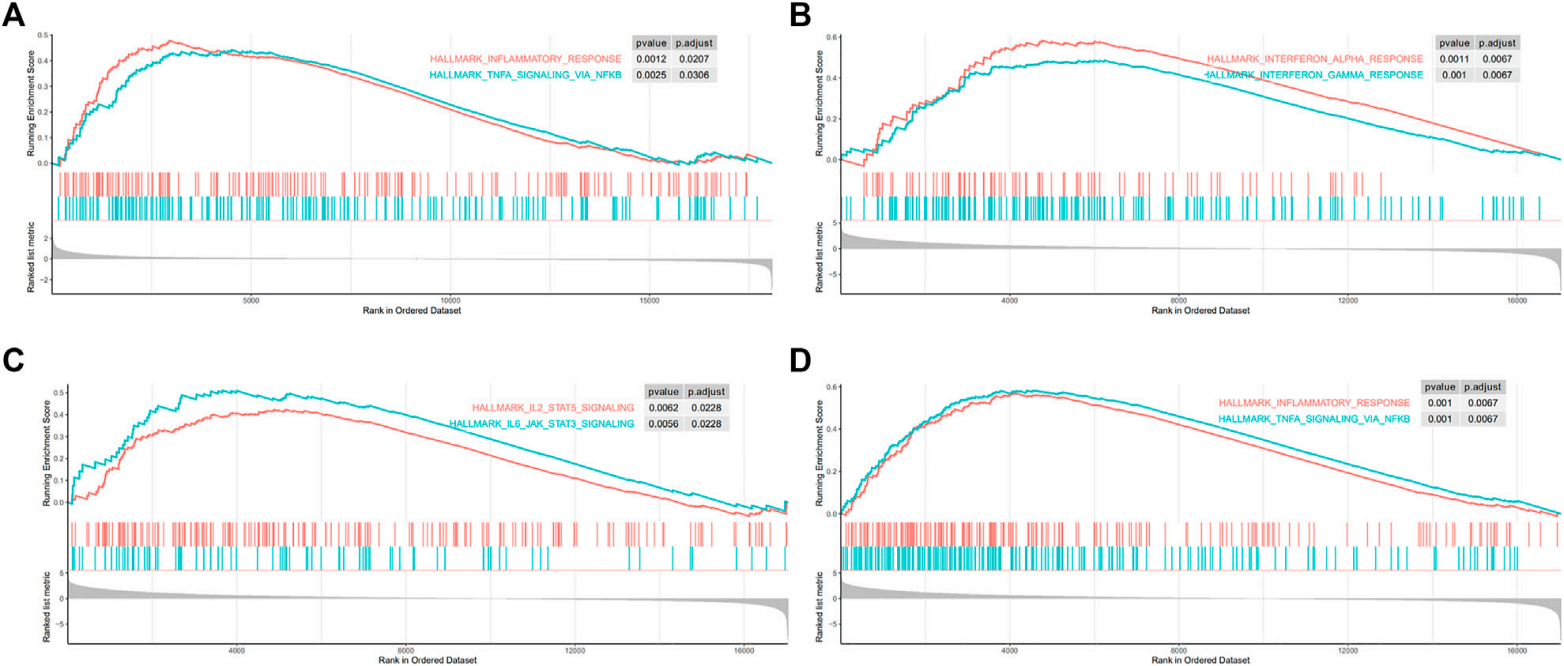
Gene set enrichment analysis (GSEA) was performed using the Hallmark gene sets **(A)** Inflammatory response pathway and TNFα-NFκB pathway were enriched in MGA mutated patients from the TCGA-LUAD cohort **(B)** IFN-α and IFN-γ pathways were enriched in advanced MGA mutated patients from stage IV TCGA-LUAD cohort **(C)** Inflammatory response pathway and TNFα-NFκB pathway were enriched in MGA mutated patients from stage IV TCGA-LUAD cohort **(D)** JAK-STAT pathways were enriched in MGA mutated patients with stage IV from the TCGA-LUAD cohort. TNFα, tumor necrosis factor-alpha; NFκB, nuclear factor kappa-B; IFN-α, interferon-alpha; IFN-γ, interferon-gamma; JAK-STAT, janus kinase-signal transducer and activator of transcription.

Gene set enrichment analysis on 50 hallmark gene sets revealed that gene sets regarding inflammatory response and tumor necrosis factor (TNFα)-nuclear factor kappa-B (NFκB) signaling were enriched in MGA-mutated samples (Figure 6A). As ICI-treated patients are usually at advanced stage, we next performed GSEA on stage IV patients of the TCGA-LUAD cohort. Results showed that a more prominent enrichment of immune activation in MGA-mutated group, including IFN-γ response, IFN-α response (Figure 6B), the janus kinase-signal transducer and activator of transcription (JAK-STAT) pathway (Figure 6C), TNFα-NFκB signaling and inflammatory response (Figure 6D).

## Discussion

The clinical application of ICIs has achieved impressive success in the treatment of advanced NSCLC. However, the majority of patients do not benefit from ICIs. Thus, biomarkers that can predict response to immunotherapy are highly needed. In this comprehensive study, we investigated whether cancer cell-intrinsic gene mutations can influence the efficacy of ICIs in non-squamous NSCLC. Our results suggested that MGA mutation was associated with better DCB, higher ORR, and improved PFS and OS. Notably, MGA mutation was predictive of survival benefit in ICI-treated population, but not in the immunotherapy-naive cohorts. And the survival benefit of MGA mutation was independent of TMB and PD-L1 expression, suggesting that MGA mutation could complement the two biomarkers in non-squamous NSCLC. Furthermore, we found that tumor immunogenicity and antitumor immunity were enhanced in non-squamous NSCLC patients with MGA mutation.

MGA, encoding MAX dimerization protein, is a tumor suppressor gene in lung cancer. It functions as a dual-specificity transcription factor that interacts with MAX and contains a T-domain DNA-binding motif (Hurlin et al., 1999). It suppresses MYC transcriptional program and inhibits E2F target genes (Hurlin et al., 1999; Ogawa et al., 2002). It is shown that inactivation at MGA is mutually exclusive with alterations of members of the switch/sucrose nonfermentable (SWI/SNF) chromatin remodeling complex and focal MYC amplification (Cancer Genome Atlas Research, 2014), suggesting that it may play a critical role in SWI/SNF-MYC functional axis (Romero et al., 2014).

Although previous studies have indicated a vital role of MGA in tumorigenesis, the effect of MGA mutations on tumor-host interactions is unclear. Our study is the first to uncover that MGA mutations in NSCLC may influence sensitivity to ICIs. Our results were not only statistically significant but also clinically meaningful. The ORR of patients with MGA mutation was 58%, which is more than a 2.5-fold increase compared to patients with MGA wild type. The survival improvement was also remarkable. MGA mutant patients had a median PFS of 21.7 months (95% CI, 12.09 to not reached), compared with 5.4 months (95% CI, 4.18–7.56) in patients with MGA wild type. The median OS of MGA mutant patients was not reached (more than 60 months) in the MGA-mutated group, vs. 12.0 months (95% CI, 10.0–16.0) in patients with MGA wild type.

The fundamental basis for response to ICIs is the immunogenicity of a tumor. Tumor antigenicity is one key determinant of tumor immunogenicity. As elevated TMB may increases the odds of generating immunogenic peptides (Schumacher and Schreiber, 2015), it is reasonable to suggest that TMB and neoantigen load are predictive biomarkers for ICI treatment. Indeed, higher best ORRs have been observed in cancers which harbor high somatic mutations, such as NSCLC and melanoma (Yarchoan et al., 2017). Nonetheless, these potential biomarkers have several limitations. Both TMB and neoantigen load are continuous variables and the standardized cutoff criteria are lacking. Although TMB has been validated in several randomized controlled trials (RCT) involving ICIs in the treatment of advanced NSCLC (Weinstock et al., 2017; Gandara et al., 2018), the sensitivity and specificity of TMB as a predictor is modest. As for neoantigen load, it is still a major challenge to specifically identify immunogenic neopeptides owing to the heterogeneity of binding affinity of the peptide-major histocompatibility complex (MHC) complex and diversity of the T-cell receptor (TCR) repertoire. Evidence showed that neoantigen burden determined by traditional methods generally performs no better than TMB in predicting the efficiency of ICIs (Rizvi et al., 2015; Hellmann et al., 2018). Interestingly, we observed that MGA mutation was positively associated with elevated TMB and higher neoantigen load. Furthermore, we also found a higher mutation frequency of major DDR pathways in MGA-mutated samples, including BER, HDR, MMR, NER, and NHEJ pathways. Alterations in DDR pathways can be a source of genomic instability and may sensitize responses to ICIs due to an elevated production of tumor-associated neoantigen. These results suggested a possibility of enhanced antigenicity in non-squamous NSCLC patients with MGA mutation. Notably, multivariable analysis in the discovery cohort, where TMB was associated with longer survival, showed that the predictivity of MGA mutation is independent of TMB.

Besides tumor antigenicity, another key determinant of tumor immunogenicity is the ability to present such antigenicity. Cytokines such as interferon (IFN), tumor necrosis factor (TNF), interleukins (IL-2, IL-6) have been acknowledged as key mediators of antitumor immunity (Kearney et al., 2017; Overacre-Delgoffe et al., 2017). IFN-α and IFN-β contribute to the antitumor activity by supporting immune cell migration, stimulation, and differentiation (Dunn et al., 2005; Diamond et al., 2011). They also increase tumor immunogenicity through the upregulation of MHC molecules on antigen-presenting cells (Keskinen et al., 1997; Gessani et al., 2014). IFN-γ is believed to be one of the critical factors determining the success of immunotherapy. While defects in the IFN-γ pathway in tumors correlate with resistance to ICIs (Gao et al., 2016), higher expression of IFN-γ-related genes were found in patients who responded to anti-PD-1 therapy in some types of cancer (Ayers et al., 2017), including NSCLC. In fact, the majority of immune responses initiated by cytokines are dependent on JAK-STAT signaling (Owen et al., 2019). Here in the present study, we observed that mutations of MGA were associated with inflammatory response, JAK-STAT pathways, and interferon pathways, which might be part of the mechanism in predicting a better outcome of non-squamous NSCLC patients harboring MGA mutations after immunotherapy.

Combination treatment with PD-1/PD-L1 and CTLA-4 blockades might provide greater anti-tumor activity than single drugs as targeting both pathways may have synergistic effects (Hellmann et al., 2017; Wei et al., 2017). Our study showed that combination therapy was associated with better DCB, higher ORR, and improved survival. Subgroup analysis indicated that MGA mutation was not associated with response to ICIs in the monotherapy group, possible due to the limited sample size. However, MGA mutation occurred exclusively in patients with DCB in the combination treatment subgroup. And MGA mutation was an independent prognostic biomarker of patient survival after adjusting treatment regimens. Further studies are needed to investigate whether patients with MGA mutation would benefit more from combination therapy.

The present study has several limitations. First, as the sample size is limited, we only include oncogenes or tumor suppressor genes that have a mutation frequency greater than 3%, which may miss some rare but important oncogenic mutations. In addition, the mutation frequency of MGA is around 7%, so only a small minority of patients were classified as MGA-mutant patients. Thus the predictive value of MGA mutation for immunotherapy should be followed-up in a larger study. Second, the retrospective design and cohort heterogeneity may introduce bias to this study. Although we used multivariate Cox proportional hazards model to adjust potential variables, unidentified factors that may influence outcomes could bias our results. In addition, the underlying molecular mechanism through which MGA mutation improve the efficacy of ICI treatment is still unknown and require future investigation.

## Conclusion

In summary, our study explored the association between genetic mutations of oncogenes and tumor suppressor genes with the efficacy of ICIs in non-squamous NSCLC patients. We found that the presence of MGA mutation was enriched in patients with durable clinical benefit, and it was associated with longer PFS and OS. In addition, we observed that MGA mutation correlated with enhanced immunogenicity and antitumor immunity. Thus, MGA mutation could serve as a novel predictive biomarker of response to ICIs in non-squamous NSCLC. Further preclinical and prospective clinical studies are warranted.

## Data Availability

Publicly available datasets were analyzed in this study. This data can be found here: All the data and material used in this study were publicly available and were retrieved from the cBioPortal for Cancer Genomics (http://www.cbioportal.org/) and the Supplementary Material of previously published studies as described in the Method section.
