# Supplementary material for: MGA Mutation as a Novel Biomarker for Immune Checkpoint Therapies in Non-Squamous Non-Small Cell Lung Cancer
Source: Front Pharmacol. 2021 Apr 13;12:625593. doi: 10.3389/fphar.2021.625593 (PMC8076956; doi:10.3389/fphar.2021.625593)
Supplement: Supplementary file 1 [file datasheet1.pdf]

Table S 1. Patient characteristics of the discovery cohort

	**Overall (N=314)**
**Age**	
Median [Min, Max]	64.0 [22.0, 92.0]
Missing	13 (4.1%)
**SEX**	
Male	135 (43.0%)
Female	179 (57.0%)
**SMOKING**	
Ever	248 (79.0%)
Never	66 (21.0%)
**TREATMENT_TYPE**	
Monotherapy	224 (71.3%)
Combination	90 (28.7%)
**TMB**	
Mean (SD)	9.12 (10.1)
Median [Min, Max]	5.70 [0.184, 91.8]
**PDL1**	
≥1%	70 (22.3%)
0%	59 (18.8%)
Missing	185 (58.9%)
**RECIST**	
CR	8 (2.5%)
PR	69 (22.0%)
SD	108 (34.4%)
PD	126 (40.1%)
NE	3 (1.0%)
**DURABLE_CLINICAL_BENEFIT**	
YES	113 (36.0%)
NO	181 (57.6%)
NE	20 (6.4%)

Abbreviations: TMB, Tumor mutational burden; PD-L1, Programmed cell death ligand-1; CR,Complete response;PR, partial response; SD, stable disease; PD, progression of disease.

Table S2. Identified oncogenes and tumor suppressor genes (n=82)

Gene Symbol	Mutation Number with DCB	Mutation Number with NCB	Total Mutation Number
ALK	7	8	15
APC	9	10	19
ARID1A	11	12	23
ARID1B	8	4	12
ARID2	10	8	18
ASXL2	8	3	11
ATM	12	13	25
ATR	6	4	10
ATRX	12	7	19
BARD1	6	4	10
BCOR	10	5	15
BRAF	6	6	12
BRCA1	6	6	12
BRCA2	9	2	11
BRIP1	7	7	14
CARD11	6	6	12
CDKN2A	8	10	18
CREBBP	10	9	19
CTNNB1	2	9	11
DDR2	8	3	11
DOT1L	8	4	12
EGFR	10	31	41
EP300	4	7	11
EPHA3	14	13	27
EPHA5	16	8	24
EPHA7	8	8	16
ERBB2	5	9	14
ERBB4	13	5	18
FAT1	19	9	28
FLT1	9	7	16
FLT4	10	5	15
GNAS	3	7	10
GRIN2A	10	8	18
HGF	10	10	20
INHBA	6	5	11
INPP4B	11	5	16
IRS1	5	5	10
KDM5C	9	2	11
KDR	7	4	11

KEAP1	25	38	63
KMT2A	10	3	13
KMT2C	22	14	36
KMT2D	12	14	26
KRAS	49	67	116
LATS2	6	4	10
MDC1	6	7	13
MED12	8	6	14
MET	8	9	17
MGA	17	6	23
NCOR1	8	5	13
NF1	12	14	26
NF2	6	4	10
NOTCH1	7	3	10
NOTCH2	8	4	12
NOTCH4	9	11	20
NSD1	4	6	10
NTRK3	9	7	16
PBRM1	3	9	12
PDGFRA	4	7	11
PGR	10	4	14
PIK3C2G	6	4	10
PIK3CA	7	11	18
PIK3CG	10	9	19
PTPRD	23	17	40
PTPRT	20	13	33
RB1	10	10	20
RBM10	12	20	32
RET	9	4	13
SETD2	10	8	18
SF3B1	6	5	11
SMAD4	3	11	14
SMARC4	19	19	38
SMO	9	4	13
STK11	17	50	67
TBX3	5	9	14
TET1	11	2	13
TET2	6	8	14
TP53	67	89	156
TP63	6	4	10
TSC2	7	3	10
TSHR	7	3	10
ZFHX3	8	7	15

Table S3. Clinical cohorts analyzed in this study

Cohort	Matthew D Hellmann	Hira Rizvi	Diana Miao	Naiyer A. Rizvi	Robert M Samstein
**Journal**	Cancer Cell	Journal of Clinical Oncology	Nature Genetics	Science	Nature Genetics
**Year**	2018	2018	2018	2015	2019
**Regimen**	Combination (PD-1 plus CTLA-4 blockade)	Anti-PD-1/PD-L1, or combined with anti-CTLA-4		Anti-PD-1	Anti-PD-1/PD-L1, or combined with anti-CTLA-4
**Cancer type**	NSCLC	NSCLC	Pan-cancer	NSCLC	Pan-cancer
**No. of nonsqumous NSCLC patients**	59	206	29	30	305
**Outcome**	ORR, PFS	ORR, PFS	ORR, PFS	ORR, PFS	OS
**NGS testing**	Whole-exome sequencing	MSK-IMPACT gene panel: 468genes;410 genes;341 genes	Whole-exome sequencing	Whole-exome sequencing	MSK-IMPACT gene panel: 468genes;410 genes;341 genes

Table S 4 . Baseline characteristics according to MGA mutation status

	MGA wild type (N=242)	MGA mutated type (N=24)	p-value	
**Age** (mean (SD))	64.16 (10.80)	65.52 (10.47)	0.56	One-way Anova
**Sex**			0.83	Fisher's exact test
Male	101 (41.7%)	11 (45.8%)		
Female	141 (58.3%)	13 (54.2%)		
**Histology**			0.67	Fisher's exact test
Adenocarcinoma	129 (53.3%)	14 (58.3%)		
Others	113 (46.7%)	10 (41.7%)		
**Smoking**			0.79	Fisher's exact test
Ever	193 (79.8%)	20 (83.3%)		
Never	49 (20.2%)	4 (16.7%)		
**Treatment type**			0.24	Fisher's exact test
Monotherapy	172 (71.1%)	14 (58.3%)		
Combination	70 (28.9%)	10 (41.7%)		
**TMB** (mean (SD))	8.18 (8.53)	15.79 (12.53)	<0.001	Man-Whitney U test

Figure Legends

Fig.S1. Enrichment of gene mutations in patients with CR/PR vs. PD in the discovery cohort (two-tailed Fisher's exact test, n = 77 patients with CR/PR, n = 126 patients with PD). Red dashed line denotes false discovery rate adjusted q = 0.05 (Fisher's exact test).

Fig.S2. Kaplan-Meier curves comparing PFS of patients with or without MGA mutations in the TCGA-LUAD cohort (A) and TCGA-LUAD cohort with stage IV patients (B).

Fig.S3. Lollipop plot shows the distribution of MGA mutations in the ICI-treated cohort and TCGA-LUAD cohort.

Fig.S4. Association of MGA Mutation with DNA damage repair gene mutations in MSKCC ICI-treated cohort (A) and MSKCC non-ICI-treated cohort (B).

Fig.S5. Association of MGA Mutation with relative abundance of infiltrated immune cell by CIBERSORT in the TCGA-LUAD cohort. Gene expression data were uploaded to the CIBERSORTx web portal (https://cibersortx.stanford.edu/), with batch correction performed and permutation number setting to 1000 for significance analysis. *$p<0.05$(Mann-Whitney U test).

Fig. S1

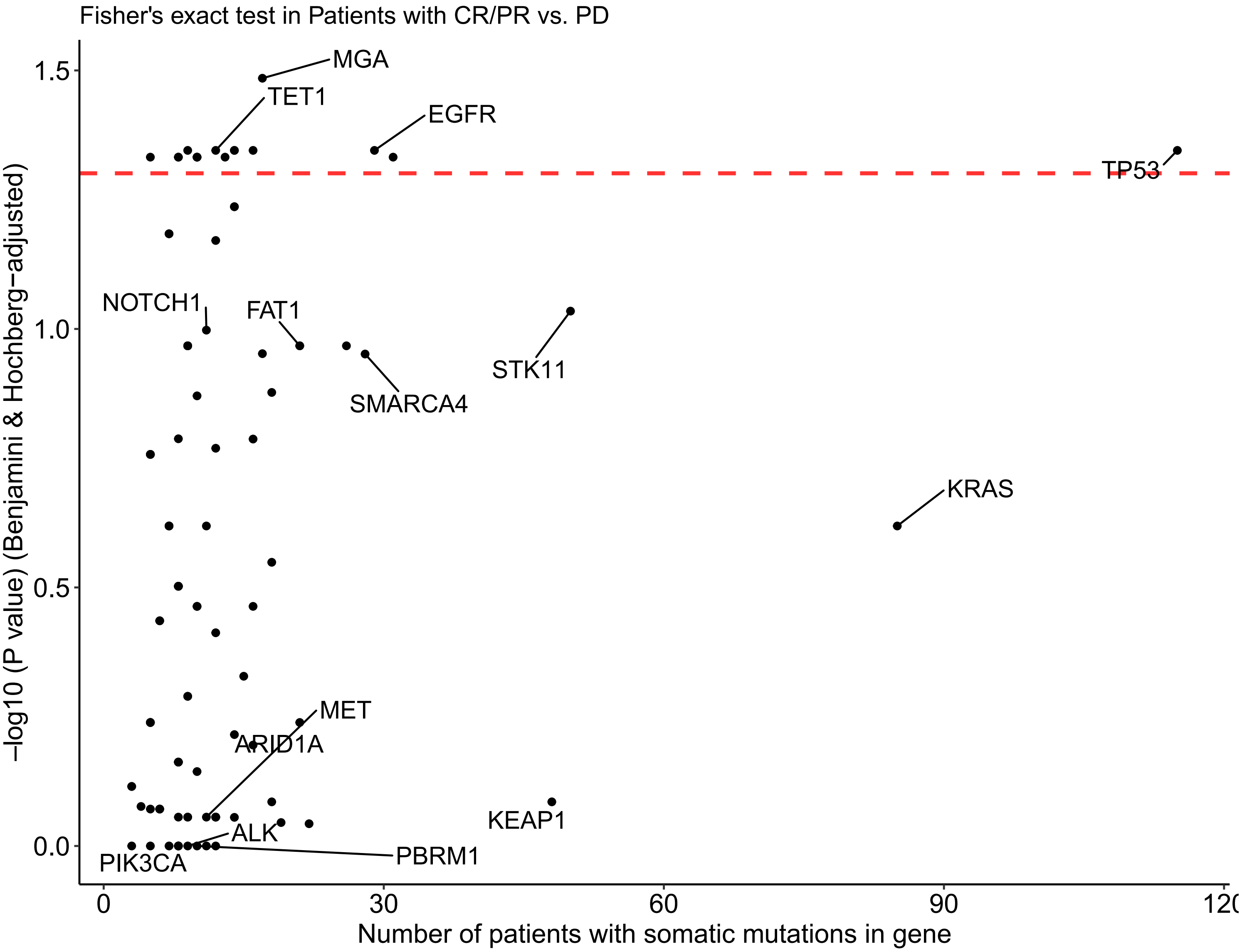

Fisher's exact test in Patients with CR/PR vs. PD
−log10 (P value) (Benjamini & Hochberg−adjusted)
1.5
1.0
0.5
0.0
MGA
TET1
EGFR
TP53
NOTCH1
FAT1
STK11
SMARCA4
KRAS
MET
ARID1A
KEAP1
ALK
PBRM1
PIK3CA
0
30
60
90
Number of patients with somatic mutations in gene

Fig. S2

A

B

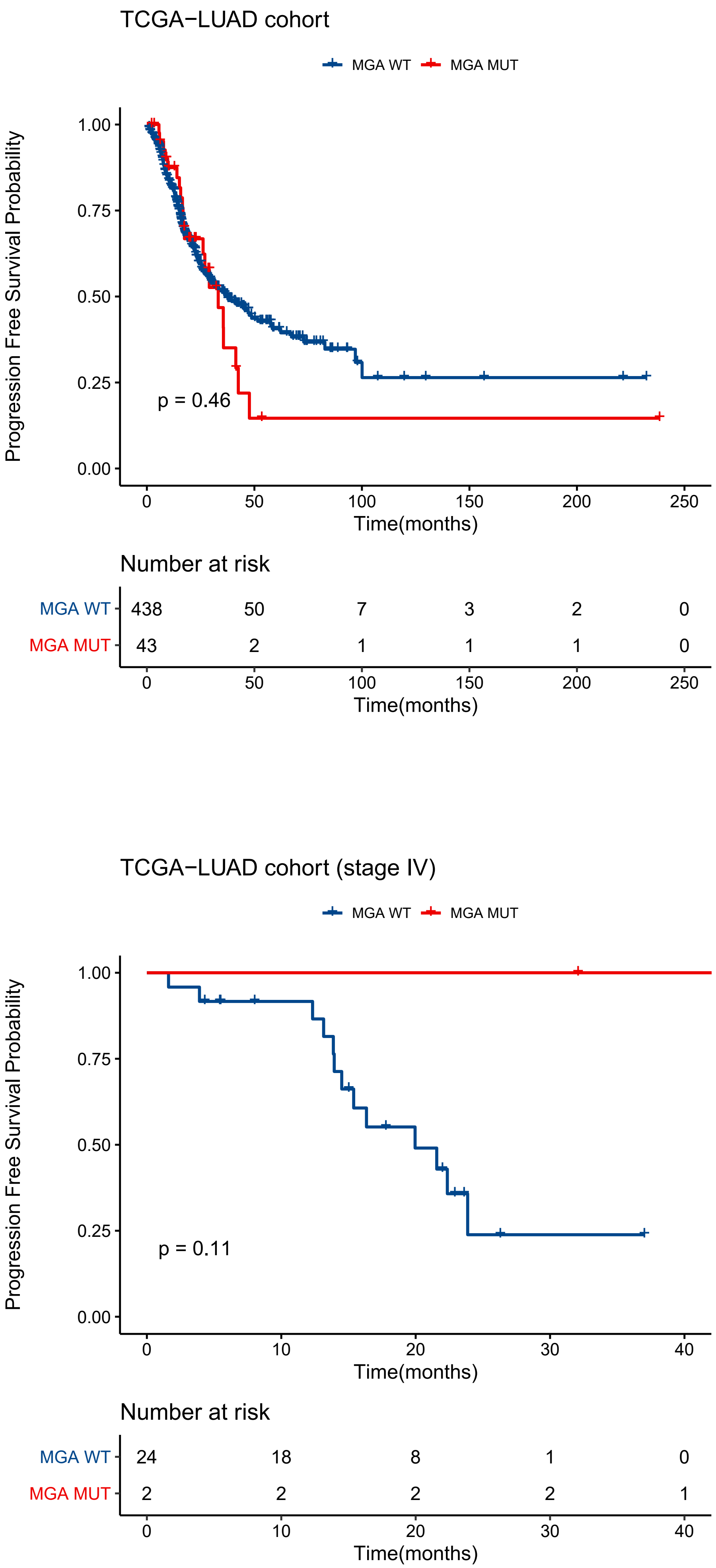
TCGA−LUAD cohort
MGA WT
MGA MUT
Progression Free Survival Probability
1.00
0.75
0.50
0.25
0.00
p = 0.46
0
50
100
150
200
250
Time(months)
Number at risk
MGA WT 438 50 7 3 2 0
MGA MUT 43 2 1 1 1 0
Time(months)
TCGA−LUAD cohort (stage IV)
MGA WT
MGA MUT
Progression Free Survival Probability
p = 0.11
0
10
20
30
40
Time(months)
Number at risk
MGA WT 24 18 8 1 0
MGA MUT 2 2 2 2 1
Time(months)

Fig. S3

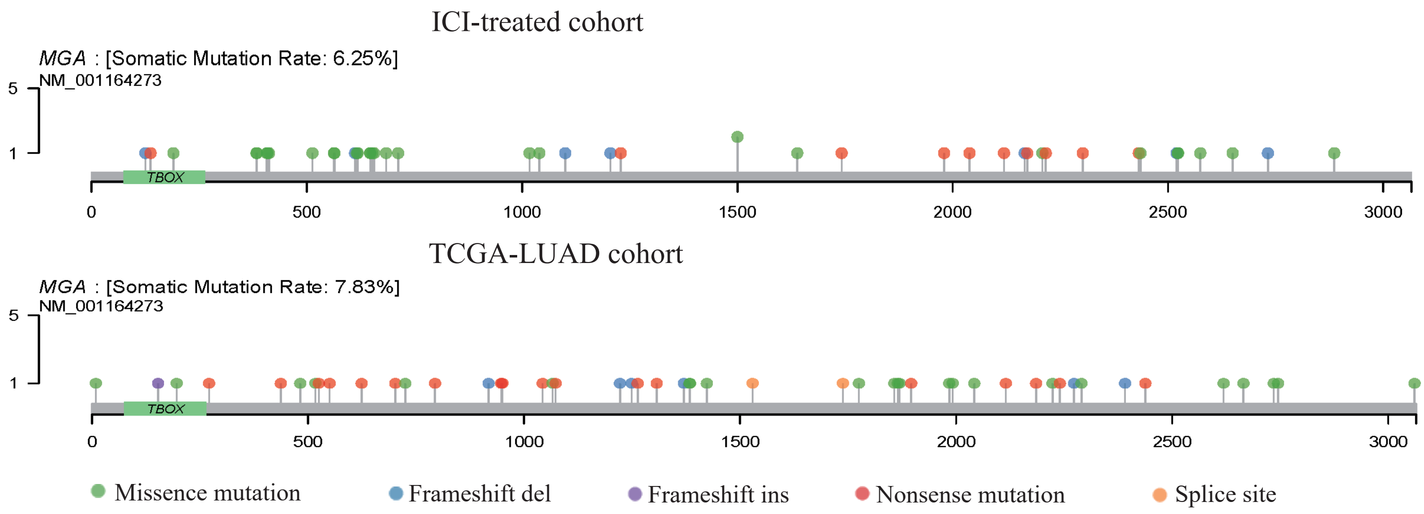
ICI-treated cohort
MGA : [Somatic Mutation Rate: 6.25%]
NM_001164273
5
1
TBOX
0
500
1000
1500
2000
2500
3000
TCGA-LUAD cohort
MGA : [Somatic Mutation Rate: 7.83%]
NM_001164273
5
1
TBOX
0
500
1000
1500
2000
2500
3000
Missence mutation
Frameshift del
Frameshift ins
Nonsense mutation
Splice site

Fig. S4

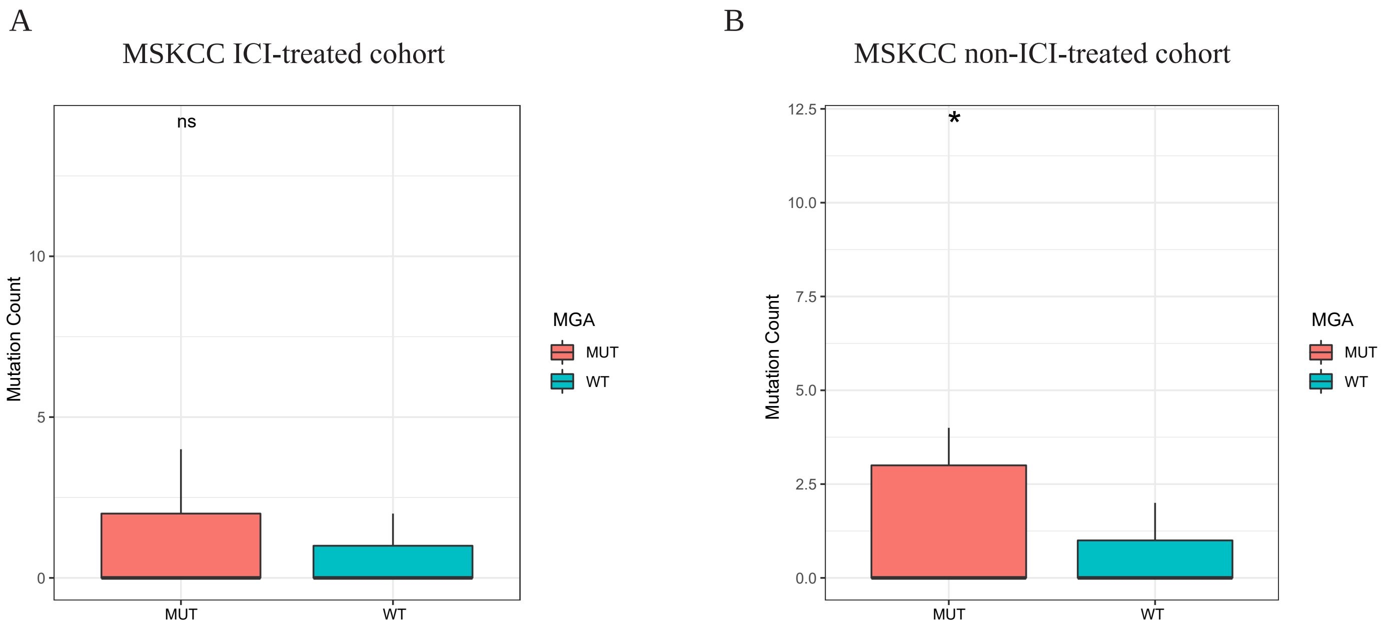
A
MSKCC ICI-treated cohort
ns
Mutation Count
0
5
10
MUT
WT
MGA
MUT
WT
B
MSKCC non-ICI-treated cohort
*
Mutation Count
0.0
2.5
5.0
7.5
10.0
12.5
MUT
WT
MGA
MUT
WT

Fig. S5

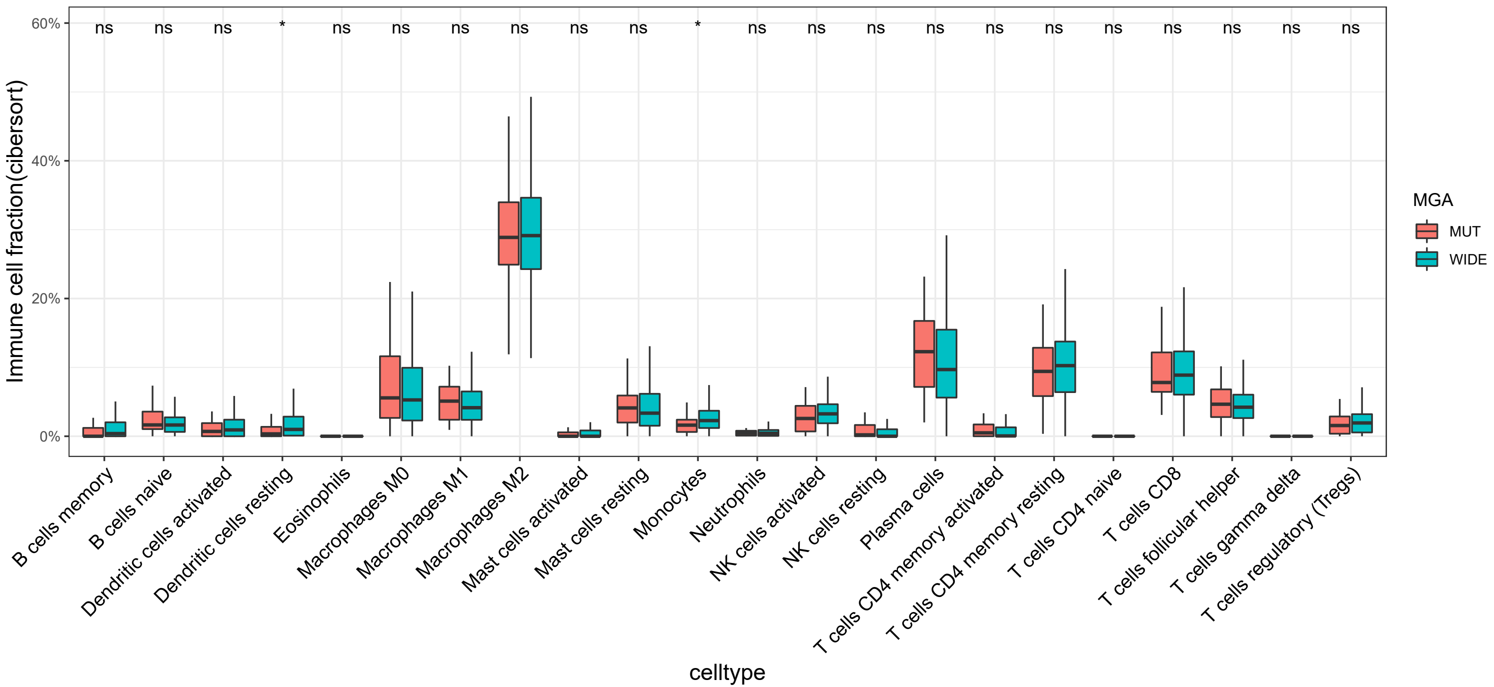

Immune cell fraction(cibersort)
60%
40%
20%
0%
ns ns ns * ns ns ns ns ns ns * ns ns ns ns ns ns ns ns ns ns ns
B cells memory
B cells naive
Dendritic cells activated
Dendritic cells resting
Eosinophils
Macrophages M0
Macrophages M1
Macrophages M2
Mast cells activated
Mast cells resting
Monocytes
Neutrophils
NK cells activated
NK cells resting
Plasma cells
T cells CD4 memory activated
T cells CD4 memory resting
T cells CD4 naive
T cells CD8
T cells follicular helper
T cells gamma delta
T cells regulatory (Tregs)
celltype
MGA
MUT
WIDE
